# Mammographic Findings after Intraoperative Radiotherapy of the Breast

**DOI:** 10.1155/2012/758371

**Published:** 2012-02-26

**Authors:** Ronald Rivera, Virginia Smith-Bronstein, Sylvia Villegas-Mendez, Jessica Rayhanabad, Pulin Sheth, Afshin Rashtian, Dennis R. Holmes

**Affiliations:** ^1^Los Angeles Center for Women's Health, 1513 South Grand Avenue, Suite 400, Los Angeles, CA 90015, USA; ^2^University of Southern California Keck School of Medicine, Keith Administration, 1974 Zonal Avenue, Los Angeles, CA 90033, USA; ^3^Division of Surgical Oncology, USC Kenneth Norris Comprehensive Cancer Center, University of Southern California Keck School of Medicine, 1441 Eastlake Avenue, Los Angeles, CA 90033, USA; ^4^Department of Radiology, USC Kenneth Norris Comprehensive Cancer Center, University of Southern California Keck School of Medicine, 1441 Eastlake Avenue, Los Angeles, CA 90033, USA; ^5^Department of Radiation Oncology, USC Kenneth Norris Comprehensive Cancer Center, University of Southern California Keck School of Medicine, 1441 Eastlake Avenue, Los Angeles, CA 90033, USA

## Abstract

Intraoperative Radiotherapy (IORT) is a form of accelerated partial breast radiation that has been shown to be equivalent to conventional whole breast external beam radiotherapy (EBRT) in terms of local cancer control. However, questions have been raised about the potential of f IORT to produce breast parenchymal changes that could interfere with mammographic surveillance of cancer recurrence. The purpose of this study was to identify, quantify, and compare the mammographic findings of patients who received IORT and EBRT in a prospective, randomized controlled clinical trial of women with early stage invasive breast cancer undergoing breast conserving therapy between July 2005 and December 2009. Treatment groups were compared with regard to the 1, 2 and 4-year incidence of 6 post-operative mammographic findings: architectural distortion, skin thickening, skin retraction, calcifications, fat necrosis, and mass density. Blinded review of 90 sets of mammograms of 15 IORT and 16 EBRT patients demonstrated a higher incidence of fat necrosis among IORT recipients at years 1, 2, and 4. However, none of the subjects were judged to have suspicious mammogram findings and fat necrosis did not interfere with mammographic interpretation.

## 1. Background/Context

Recent publication of a randomized trial comparing the efficacy and safety of targeted intraoperative partial breast radiotherapy (IORT) and standard postoperative whole breast external bream radiotherapy (WB-EBRT) has drawn worldwide attention to the promise of IORT for reducing the local recurrence rate and treatment burden following breast conserving surgery [1]. This international, multicenter study of 2233 women with early-stage invasive breast cancer comes on the heels of several nonrandomized studies demonstrating comparable efficacy and safety between standard postoperative WB-EBRT using different IORT approaches [2–5]. Collectively, these findings have laid the foundation for the emergence of IORT as an alternative to WB-EBRT for the conservative management of early-stage invasive breast cancer.

The current worldwide standard for the management of invasive and noninvasive breast cancer is mastectomy or breast-conserving surgery followed by WB-EBRT when local excision can be achieved with acceptable cosmesis [6–11]. WB-EBRT is commonly prescribed at a total physical radiation dose of 50 Gray (Gy) administered in 25 daily fractions of 2 Gy each, typically followed by 5 daily 2 Gy fractions administered to the tumor bed as a radiation “boost” [12]. IORT is an alternative method of breast radiotherapy in which a complete course of breast radiotherapy is administered at the time of primary tumor resection or reexcision. IORT administered to the tumor bed may also substitute for an external beam boost when the patient is expected to receive WB-EBRT [13, 14]. As a form of accelerated partial breast radiotherapy (APBI), the purpose of IORT is to administer a complete course of radiotherapy to a predefined distance beyond the surgical margins in a single fraction of approximately 21 Gy [1]. Not only does this greatly increase the feasibility and efficiency of breast radiotherapy, but also the targeted irradiation of the index quadrant minimizes collateral radiation injury to the nonindex quadrants and more distant tissues (e.g., skin, ribs, lung, heart) through the use of local shielding, collimators, and skin retractors. 

**Figure 1:**
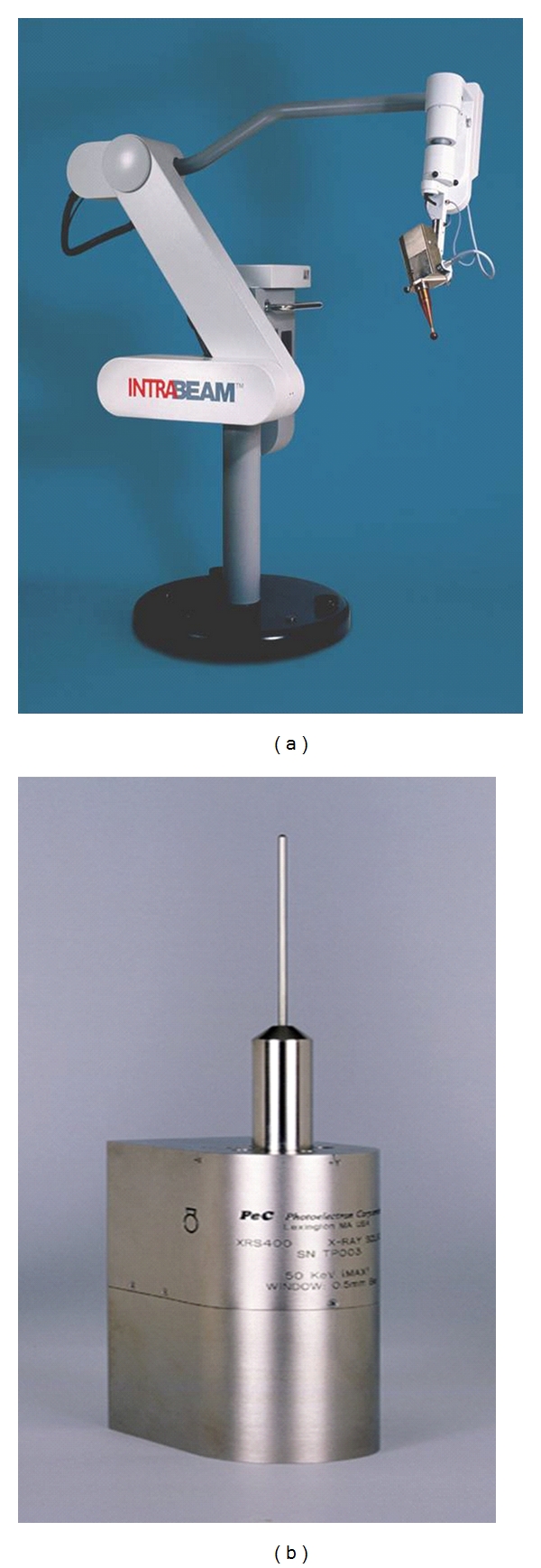
The Intrabeam System (a) and X-ray source (b).

Semiannual or annual diagnostic mammography is routinely utilized to screen for local recurrence of breast cancer after breast-conserving surgery. Since the majority of these recurrences appear within the first 5 years postoperatively, a mammographer's challenge is to distinguish between the seminal parenchymal changes associated with cancer recurrence and the usual treatment-related changes that may evolve in the initial years following breast-conserving therapy [10]. The growing popularity of APBI, in general, and IORT, in particular, has led physicians to question whether administration of focused, high-dose radiation to the tumor bed might produce parenchymal changes that could interfere with mammographic surveillance of cancer recurrence or lead to excessive diagnostic or invasive procedures to evaluate mammographic findings. There are only a few published papers describing the radiographic appearance of the breast following IORT [14–18]. With each, investigators observed varying degrees of fat necrosis and parenchymal scarring, likely related to the technique of IORT delivery. In this investigation, we examined the mammographic features of a subset of participants in a published randomized clinical trial comparing WB-EBRT and low-energy IORT delivered using the Intrabeam System (Carl Zeiss Meditec, Inc., Oberkochen, Germany, Figure 1).

## 2. Design

**Figure 2:**
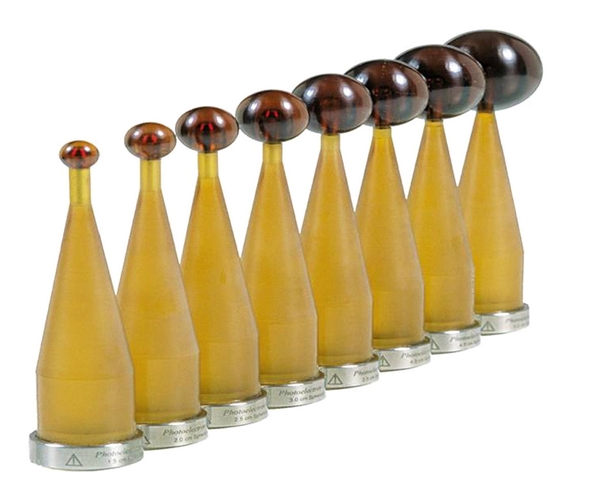
Fixed diameter (2–5 centimeter) spherical applicators for the Intrabeam System.

A retrospective analysis was performed to identify, quantify, and compare the mammographic findings among participants in the TARGIT Trial, a prospective, randomized, controlled clinical trial comparing targeted single fraction IORT to conventional multiple fraction postoperative WB-EBRT for the treatment of early-stage invasive breast cancer. The TARGIT trial is a multicenter, international trial of over 2200 participants enrolled at 28 facilities in 9 countries. The present study is limited to TARGIT Trial participants enrolled at the University of Southern California Kenneth J. Norris Comprehensive Cancer Center in Los Angeles, California. Participants randomized to the WB-EBRT treatment arm received a 5-week course of daily radiotherapy (45–50 Gy) on stationary linear accelerators at the Kenneth J. Norris Cancer Center or at radiation oncology centers in their home communities. WB-EBRT recipients also received a 5-day external beam boost (10 Gy) at the discretion of their treating radiation oncologist. All patients randomized to the IORT group received IORT to the tumor bed at the time of initial lumpectomy or at the time of margin reexcision using the Intrabeam System. The Intrabeam is a low-energy (50 kV) radiotherapy system that uses X-rays to deliver a physical dose of radiation (20 Gy) to the surgical margin using multiple fixed-diameter (Figure 2) or expandable applicators to conform the breast tissue around the X-ray source [19]. Interim 4-year results of the TARGIT Trial showed statistically equivalent local control and safety in the IORT and WB-EBRT treatment groups (local rate of recurrence: 1.2% versus 0.95%, *P* = 0.41) [1].

All study participants were followed semiannually with clinical breast examination, diagnostic mammography of the ipsilateral breast, and annual mammography of the contralateral breast. Mammograms from these followup evaluations were selected for review at the 1-, 2-, and 4-year intervals after surgery. Analysis was limited to participants with a minimum of 4 years postoperative followup to allow time for resolution or improvement of acute and subacute treatment-related parenchymal changes that could affect mammographic interpretation. Mammograms from each time point were reviewed by a mammographer with more than 10 years of experience in breast imaging. The mammographer was blinded to the type of breast radiotherapy received by each study participant. Standard mediolateral oblique views and craniocaudal views of the affected and unaffected breasts were evaluated for the presence of 6 predefined mammographic features (architectural distortion, skin thickening, skin retraction, calcification, fat necrosis, and mass density) which were assessed, in part, by comparing each patient's treated breast to her contralateral untreated breast. These data points were selected based on published literature identifying each as a potential sequela of breast surgery and/breast radiotherapy [20–24]. A BI-RADS score was generated using the American College of Radiology analysis criteria [25]. The following participants were excluded from this analysis: (1) patients who did not have available mammograms from each of the specified time points; (2) patients who received mastectomy after prior IORT; (3) patients who were referred for WB-EBRT after prior IORT due to positive nodes or positive surgical margins. An analysis of variance with repeated measures was performed to determine if the incidence of each outcome measure at years 1, 2, and 4 after surgery was significant by treatment group and across each time point. The study was approved by the institutional review board.

## 3. Results

A total of 49 patients were treated in the TARGIT trial at the University of Southern California Kenneth J. Norris Comprehensive Cancer Center between April 2005 and January 2010. Of these patients, 19 were excluded from central review of imaging: 12 patients had not reached 4 years of followup by the time of analysis, 3 patients did not have mammograms available for central review, and 4 patients underwent completion mastectomies. The remaining 30 TARGIT trial patients had completed more than 4 years followup with central review of their surveillance mammograms. 90 sets of mammograms were analyzed for 14 (47%) patients receiving IORT and 16 (53%) patients receiving WB-EBRT. The median age of patients participating in the study was 58 years (range 40–81). The median tumor size was 15 mm (range 3–25) in the IORT cohort and 16 mm (range 7–38) in the WB-EBRT cohort (Table 1).

**Figure 3:**
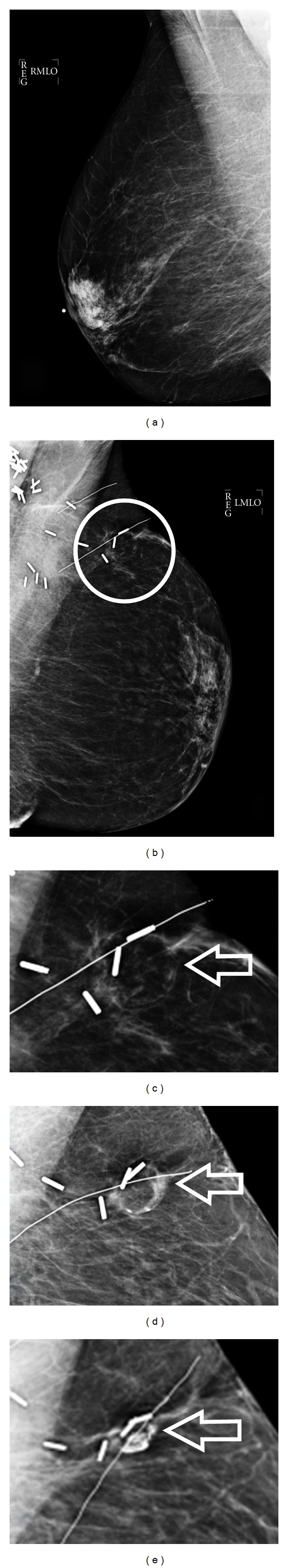
(a and b): 1-year postoperative bilateral mammograms following IORT showing the development of fat necrosis (circle). (c, d, e): Mammograms of same patient showing fat necrosis with oil cyst formation at 1 year post-IORT (c), development of a calcifying rim at 2 years post-IORT (d), and resolution of oil cyst with residual calcifications at 4 years post-IORT(e).

We compared the incidence of each mammographic finding in the treatment cohorts using univariate testing by repeated measures ANOVA. For the between-subjects measurements, no statistically significant differences were found in the incidence of architectural distortion (*P* = 0.21), skin thickening (*P* = 0.50), skin retraction (*P* = 0.39), calcifications (*P* = 0.08), or mass density (*P* = 0.20). However, the incidence of fat necrosis was observed to be significantly higher in the IORT cohort (*P* = 0.05) (Table 2(a), Figure 3). Analysis of the evolution of mammographic features over the 4-year period revealed no statistically significant temporal differences between the two treatment groups for the timing of feature incidence or resolution. This would indicate that there were no treatment-based differences in the appearance or resolution of architectural distortion, retraction, calcifications, fat necrosis, or mass density over time. However, a trend of increased skin retraction was observed in the WB-EBRT group (*P* = 0.06, Table 2(b)).

Table 3 shows the incidence of six mammographic features detected within each arm of the study during the 4-year followup period. Architectural distortion, skin thickening, skin retraction, and calcifications were commonly detected in both arms of the study, with fat necrosis and mass density occurring less often. Nonetheless, the incidence of fat necrosis was 3 times higher in the IORT group. In all cases, areas of fat necrosis acquired a characteristically benign appearance with the passage of time and with the formation of macrocalcifications. All of the mammographic findings, including the areas of fat necrosis, were judged to be radiographically benign, and none were given a BI-RADS categorization of 4 or 5 to indicate the need for a diagnostic core needle biopsy to rule out recurrent malignancy.

## 4. Discussion

The growing use of APBI, in general, and IORT, in particular, has the potential to introduce new challenges in the mammographic surveillance of breast cancer recurrence. In one of the first studies to address this concern, Sala et al. compared 45 patients who received IORT (20–24 Gy) using a high-dose rate linear accelerator (Precise Radiotherapy System, Elekta Oncology, Inc., Stockholm, Sweden) to 45 patients who received WB-EBRT (50 Gy + 10 Gy boost) and found that the IORT group demonstrated a greater degree of architectural distortion, breast edema, and fat necrosis, each becoming increasingly prominent over the 24 months period after radiotherapy [15]. Ruch et al. studied 54 patient who received IORT alone (*n* = 14, 20 Gy) or IORT boost (*n* = 40, 20 Gy boost followed by 46 Gy WB-EBRT) using the low-energy Intrabeam System and similarly observed a higher incidence of fat necrosis and prolonged parenchymal scarring in the IORT boost group at 3 years of followup compared to a nonrandomized cohort of 48 patients receiving WB-EBRT alone (55 Gy) [18]. In contrast, Kuzmiak et al. compared 32 IORT patients treated with the high-dose rate Mobetron system (Intraop Medical, Sunnyvale, CA) with a random sample of 32 patients treated with WB-EBRT and reported significantly more breast edema and surgical scaring in the WB-EBRT group at 1 year of followup but did not disclose the incidence of fat necrosis in either group [16]. Similar to Della Sella and Ruch, Wasser et al. detected a higher incidence of fat necrosis and parenchymal scarring following an IORT boost (20 Gy followed by 46 Gy WB-EBRT) over a 2-year period compared to a nonrandom contemporary sample of patients treated with WB-EBRT with or without an external beam boost (56–66 Gy) [14]. In the current investigation, we performed a retrospective review of mammographic findings among participants in the prospective, randomized controlled TARGIT Trial who received either low-energy-targeted IORT alone (20 Gy) or WB-EBRT with or without an external beam boost (50–60 Gy). Unlike the studies referenced above, we did not observe a statistically significant difference between the IORT and WB-EBRT groups in the 1-, 2-, and 4-year incidence of architectural distortion (i.e., parenchymal scarring). We also did not detected a statistically significant difference between the two treatment groups in the 1-, 2-, and 4-year incidence of dystrophic calcifications, skin retraction, or mass density, though there was a trend toward a higher incidence of skin thickening (i.e., breast edema) in the WB-EBRT group as similarly observed by Kuzmiak. Our most significant finding was the nearly 3-fold higher incidence of fat necrosis in the IORT group. This finding was consistent with the higher incidence of IORT-related fat necrosis reported by Sala, Wasser, and Ruch. In contrast with Ruch et al., fat necrosis did not confound the interpretation of the mammograms in the IORT group and none of the subjects were given a BI-RADS score of 4 or 5 to indicate the presence of a mammographic finding warranting a diagnostic core needle biopsy. 

Fat necrosis following breast surgery or breast radiotherapy results from tissue devitalization leads to liquefactive necrosis, formation of oil cysts (liquefied fat) or fibrous scars, and frequent aggregation of spherical lucent centered macrocalcifications or “eggshell” calcifications surrounding an oil cyst. In addition to the present study, three out of four of the referenced studies recognized fat necrosis as a common finding in patients treated with either IORT alone or IORT administered as a boost, with increased fat necrosis being observed after high- and low-energy treatments. While it is reasonable to attribute the higher incidence of fat necrosis after IORT to the delivery of a focal dose of radiation to the tumor bed, a number of other factors may contribute to the posttreatment artifacts observed in IORT-treated patients. Foremost among these are the surgical methods and IORT techniques used to prepare the breast for IORT which typically involves circumferential dissection of the skin edges away from the underlying breast parenchyma, placement of conforming sutures to stabilize the surgical margins during the radiotherapy treatment, and possible dissection of the breast parenchyma off the chest wall to create a space for placement of a radiation barrier. Use of a radiation barrier is mandatory when the high-dose rate systems are used (e.g., Mobetron or Novac7) but optional with the Intrabeam due to rapid attenuation of the low-energy dose by normal tissues. In the treatment period covered in this investigation, the surgeon routinely used circumferential skin flap dissection, conforming suture placement, and radiation barrier placement to prepare the breast for IORT, and any of these steps could have contributed to the higher incidence of fat necrosis observed in the IORT cohort. In more recent years, we have limited the extent of skin dissection to minimize the risk of fat necrosis, and radiation barriers are now used more selectively based on recent data confirming marked dose attenuation. Radiographic followup of more recently treated patients will determine how these technique modifications influence mammographic findings in IORT-treated patients. The reference articles did not provide sufficient data to determine how IORT administered alone or as a boost impacts the incidence of fat necrosis.

Postoperative mammographic findings after IORT might also be influenced by the techniques used by surgeons to close the surgical cavity after breast surgery. Partial thickness closure of the lumpectomy cavity might have contributed to the higher incidence of persistent hematoma/seromas or oils cysts observed in the referenced studies, whereas full-thickness closure of the surgical cavity among IORT and WB-EBRT recipients likely contributed to a lower incidence of persistent seroma in the present study. Interestingly, a significantly higher incidence of recurrent seromas was reported among IORT recipients in the TARGIT Trial (IORT 2.1% versus WB-EBRT 0.8%, *P* = 0.012). However, the trial did not document the manner of wound closure nor did it track posttreatment mammogram findings to allow assessment of the long-term impact of wound closure techniques. Asymptomatic and symptomatic seroma formation is a commonly reported sequela of intracavity brachytherapy (e.g., MammoSite), the most widely used form of breast APBI, but seroma formation does not appear to significantly impair the radiographic surveillance of cancer [26].

There are several important limitations of the present study. Unlike the Della Sella and Wasser studies which reviewed multiple imaging modalities, we only examined the mammographic features of breasts treated with IORT or WB-EBRT. Had we expanded our assessment to include breast ultrasound or breast magnetic resonance imaging, we may have observed additional findings that could have affected our interpretation of the impact of IORT on mammography. However, since neither ultrasound nor MRI were routinely performed during the postoperative surveillance of study participants, we are unable to characterize the sonographic or MRI manifestations of breast IORT.

Another limitation of our study is the relatively small sample size of 30 patients. Since we limited the retrospective review to only those patients with a minimum of 4 years postoperative followup, the number of patients in our study was smaller. Despite these limitations, our study has several key strengths that make it an important contribution to the body of literature on postradiotherapy breast cancer surveillance following APBI. First, this 4-year radiographic followup of study participants provides the most long-term imaging evaluation involving any form of IORT of the breast. Restricting the study to patients with 4-year followup allowed us to analyze the temporal influence of surgery and breast radiotherapy on mammographic interpretation. Although the incidence of symptomatic seromas requiring aspiration might in theory be higher in the immediate postoperative period among IORT recipients, the fact that none of our patients exhibited suspicious mammographic findings provides reassurance that low-energy IORT is unlikely to increase the need for invasive diagnostic biopsies to rule out recurrent malignancy. Another important strength of this study was our ability to limit selection and treatment bias among study participants. Selection bias was limited by restricting the analysis to participants in a prospective, randomized clinical trial and treatment bias was minimized by the fact that both cohorts were treated by a single surgeon who routinely performed full-thickness oncoplastic wound closure following tumor resection.

An additional strength of the present study was the investigator's use of an image assessment scorecard to evaluate the presence, extent, or absence of 6 distinct mammographic findings, using each patient's contralateral breast as an internal control. Although not all patients obtained followup mammograms at USC where digital mammograms are routinely performed, mammographic interpretation was facilitated by scanning all analog films to digital media prior to interpretation. Observer bias was minimized by having all mammograms retrospectively reviewed by a blinded central mammographer instead of using interpretations provided by other mammographers who might have been aware of the radiotherapy treatment received.

All studies were originally read as BIRADS 2 or 3, and the retrospective review performed as part of this study confirmed those interpretations. The study radiologist was not necessarily the same radiologist who performed the original interpretation. While this does not quite constitute a double-reading, the fact that none of the study subjects were assessed to have BIRADS 4 or 5 mammograms (either originally or retrospectively) underscores the most important finding in this study that IORT did not increase the need for diagnostic core needle biopsies.

## 5. Conclusion

IORT is a form of APBI that has been shown to be equivalent to conventional WB-EBRT in terms of local cancer control and safety. In this blinded review of 90 sets of mammograms from 14 IORT and 16 WB-EBRT patients, we found a significant increase in the incidence of fat necrosis in the IORT cohort compared to patients treated with WB-EBRT. However, none of the study participants were observed to have suspicious mammographic findings. Based on these findings, we conclude that IORT and WB-EBRT have an almost equivalent potential to affect mammographic interpretation and that IORT does not disproportionately impair mammographic surveillance for breast cancer recurrence. Further studies will be needed to determine how limiting the extent of skin and parenchymal dissection influences the incidence fat necrosis after IORT.

## Figures and Tables

**Table 1:** Summary of patient age and tumor size.

	IORT	WB-EBRT	Total
Patient total (*n*)	14	16	30
Median age	59 (range 40–73)	57 (range 40–81)	58 (range 40–81)
Median size (mm)	15 (range 3–25)	16 (range 7–38)	15 (range 3–38)

**Table:** (a)

Treatment × finding	WB-EBRT (*n* = 16)	IORT (*n* = 14)	*F*	*P*-value
*Architectural distortion *				
Year 1	9	9		
Year 2	6	9		
Year 4	5	8		

Total	**20**	**26**	**1.65**	**0.21 **

*Skin thickening *				
Year 1	8	4		
Year 2	7	5		
Year 4	7	6		

Total	**22**	**15**	**0.47**	**0.5 **

*Retraction *				
Year 1	4	9		
Year 2	8	7		
Year 4	8	7		

Total	**20**	**23**	**0.76**	**0.39 **

*Calcifications *				
Year 1	0	1		
Year 2	2	6		
Year 4	3	5		

Total	**5**	**12**	**3.24**	**0.08 **

*Fat necrosis *				
Year 1	0	3		
Year 2	2	5		
Year 4	1	5		

Total	**3**	**13**	**4.2**	**0.05 **

*Mass density *		
Year 1	1	1
Year 2	3	0
Year 4	0	0

Total	**4**	**1**	**1.7**	**0.2**

**Table:** (b)

Treatment × time	*F*	*P*-value
Architectural distortion	0.87	0.43
Skin thickening	0.58	0.56
Retraction	3.01	0.06
Calcifications	1.06	0.36
Fat necrosis	0.32	0.73
Mass density	1.72	0.19

**Table 3:** Number and percentage of patients presenting with each mammographic finding in at least one screening mammogram.

Mammographic finding	IORT (*n* = 14)		WBEBRT (*n* = 16)		Total (*n* = 30)	
Architectural distortion	8	57.1%	9	56.2%	17	56.7%
Skin thickening	7	50.0%	10	62.5%	17	56.7%
Retraction	6	42.9%	10	62.5%	16	53.3%
Calcification	6	42.9%	4	25.0%	10	33.3%
Fat necrosis	5	35.7%	2	12.5%	7	23.3%
Mass density	1	7.1%	4	25.0%	5	16.6%
